# The 2018 AAP/EFP classification of periodontal diseases, a focus on “risks” as a faux ami and language gone on holiday

**DOI:** 10.1002/cre2.257

**Published:** 2019-10-30

**Authors:** Asbjorn Jokstad

1

The research and clinical management of periodontal diseases engage some of our brightest colleagues. Perhaps this is because of the intellectual challenges of understanding and managing a complex disease consisting of an interplay of genetic, environmental, and lifestyle factors, in parallel with dysbiosis, biopsychosocial elements, and a possible bidirectional relationship with systemic health, all compounded by ageing effects on humoral immune responses.

New research findings dictate regular revisions of classification systems for the periodontal diseases (American Academy of Periodontology [AAP], 1989; AAP, 1999). Novel scientific evidence, particularly within genomics and proteomics, prompted the AAP and the European Federation of Periodontology (EFP) in 2015 to reappraise the science and create a new classification scheme (Caton, Armitage, Berglundh, et al., 2018). The steering committee invited recognized content experts to compile systematic reviews for critical appraisal and discussions in a workshop held in November 2017. The workshop proceedings were presented on June 2018 at the EFP conference in Amsterdam, which coincided with publications of the SRs and consensus reports in open‐access supplements of the official journals of EFP (Journal of Clinical Periodontology) and AAP (Journal of Periodontology).

My impression after leaving the 2018 EFP conference, where the new classification was first presented publicly, and having listened to several presentations of the new classification scheme in subsequent seminars and conferences is that the new system is readily understood and accepted by many, but the staging and especially grading element of periodontitis confuse some. I believe the staging of periodontitis as a combination of severity and treatment complexity seems logical. The severity stages are determined following measurements of the clinical attachment loss interdentally or radiographic bone loss, which are diagnostic tests with relatively good interrater and intrarater reliability. A positive element of the definition of the four stages of treatment complexity is that the wordings denote operational thresholds indirectly. Of course, the need for a Stage I can be disputed between believers of respectively essentialistic versus nominalistic approaches to diagnostics of diseases in general and periodontitis in special (Scadding, 1996). I claim impartiality since I am not a content expert on the sensitivity and specificity of the latest diagnostics tests for periodontal diseases based on salivary or gingivocrevicular biomarkers. The confusion and scepticism towards the new classification system is more about the proposal for grading periodontitis based on guessing on a “high, moderate, or low risk of progression” (Caton et al., 2018). Firstly, it is unfortunate that the proofreader did not correct this snippet in the draft to either “high, moderate, or low rate of progression,” alternatively to “high, moderate, or low risk of further progression,” to conform to the consensus report from the pertinent workgroup (Papapanou et al., 2018).

Regardless, the focus on the term “risk” is unfortunate, because both laypersons and professionals use this ill‐defined and ambiguous term in everyday language. The word evokes different emotional responses, and although “risk” is a neutral term, it carries a negative connotation for many because media frequently associate “risks” with unpleasant narratives. The 2017 proceedings brim with references to “risk” in one context or another, peaking with *n* = 41 uses in one paper (Tonetti, Greenwell, & Kornman, 2018). Of the approximately 200 uses in the proceedings papers (excluding the titles in the reference lists), the majority are epidemiological‐statistical terms such as “risk factor,” “risk marker,” “risk determinant,” and “risk of bias” (Needleman et al., 2018) or “risk” for a range of stated intraoral adverse conditions (Albandar, Susin, & Hughes, 2018). However, there are several occurrences where it is apparent that the authors denote “probability,” “chance,” or “likelihood” and not “risk.” One may speculate whether there is some element of anchoring effect from citing epidemiology data where the convention is that relative event rates in different patient cohorts are described by labels such as “relative risk,” “risk ratio,” and “risk reduction.” Risk in this context is just a word and could, as well, been replaced by estimates of “odds r.” if slightly different mathematical formulas are used.

The emphasis on “risk” in the 2017 proceedings contrast the 1999 proceedings and parameters of care developed by the AAP (AAP, 2000). These documents contain the term “risk” less frequently (*n* = 56) and predominantly in context with the statistical terms “risk factor” (n=34) and “risk indicator” (*n* = 2). The other uses of “risk” in the 1999/2000 documents are in context to the likelihood of the development of adverse intraoral conditions, that is, “r. of progressive recession (of gingiva)” and “r. for implant failure” or adverse medical conditions, that is, “medical r.,” “r. for certain conditions,” “r. of cardiovascular complications,” “r. of coronary heart disease,” “r. of cerebral ischemia and non‐hemorrhagic stroke,” “r. for pre‐term low birth weight delivery,” and “at r. for infective endocarditis” and “treatment considerations for patients at r. for or with existing cardiovascular diseases.” “Risk” in the 1999/2000 documents is presented and described according to the general understanding of what a “risk factor” constitutes, that is, “any attribute, characteristic or exposure of an individual that increases the likelihood of developing a disease or injury” (WHO, 2019), alternatively “something that increases the chance of developing a disease” (National Cancer Institute, 2019a). The term was adopted in the late 50s to denote clinical and lifestyle variables that correlate positively or negatively with heart disease, while “prognostic factor” came some years later. It is essential to realize that both risk and prognostic factors are often identified in multivariate analyses of patient populations and patient cohorts are mathematical constructs and not necessarily causal factors.

In the 2017 proceedings, the use of the term “risk” is contextually correct in the well‐written and coherent consensus report made by the particular workgroup dealing with classification of periodontitis (Papapanou et al., 2018). The authors use the term in “risk factor,” and both “risk for systemic dissemination” and “risk that the disease or its treatment may negatively affect the general health of the patient” addresses new adverse health conditions. More debatable is the descriptor “risk for further progression,” which also appears in some of the other proceedings papers. The statistical term used to describe factors that influence the natural course of an established disease is “prognostic factor.” For example, “A situation or condition, or a characteristic of a patient that can be used to estimate the chance of recovery from a disease or the chance of the disease recurring (coming back)” (National Cancer Institute, 2019b). Admittedly, the medical literature contains many articles with titles containing “Risk factors for progression of … disease … ,” indicating that many scientific journals do not practise strict editorial policies on the epistemology of “risk factor.” Dental professionals, however, should be aware of the difference. One of the other workgroups adopted a variant descriptor, that is, “risk of recurrent periodontitis” (Chapple et al., 2018). However, the notion of recurrent disease is contrary to the consensus that once an individual develops periodontitis, the condition remains latent. Since the periodontitis does not disappear, it seems confusing that the same should recur, and perhaps another adjective to “recurrent” should have been preferred. Additional ambiguous terms found in other papers are, for example, “residual disease risk post‐treatment” and “risk factors for periodontitis progression” (Tonetti et al., 2018). These terms may be understandable for qualified readers, but “prognostic factors” seems more correct. The wordings may be due to carry‐over from a paper concluding that several “risk factors” for periodontitis may also predict the extent of tissue damage following periodontal therapy. Hence, some “risk factors” for periodontitis may also be considered as “prognostic factors,” although the latter term does not appear anywhere in the text (Lang, Suvan, & Tonetti, 2015).

Many of our clinical colleagues are uncomfortable with using the term “risk.” Moreover, the term is recognized as a typical *faux ami* amongst translators, and while most professionals use the term within their work domains, their interpretations differ. For example, engineers are experts on “risks” in terms of probability of flaws and possible consequences, bankers recognize “risks” of no return of their investments, traders appraise market “risks,” insurance brokers base premiums on identifiable hazards, potential perils and “risks,” liability “risk” is the bread and butter for attorneys and “due diligence” in business dealings is just another term for assessing “risks”. The list can be expanded further. Moreover, even if everyone consented to a common understanding of the term, for example, according to the International Organization for Standardization (ISO) defining “risk” as “the effect of uncertainty on objectives,” any discussions about “risks” in a clinical setting seems out of context. Few clinicians are comfortable to proceed as actuaries to explain and discuss “risks” with patients and particularly not with a coincidental professional who is acquainted with the term as it is used within their expertise domain.

I believe much confusion and reluctance towards adopting the proposed grading of periodontitis can be resolved if the focus on “risk” could be toned down. Dental professionals should instead concentrate on words that patients are more likely to grasp. Hence, instead of a focus on terms such as “risks” and “risk of progression” (of periodontitis), it would be better to operate with terms that are more familiar and neutral, such as “good, uncertain, or guarded” and “chance”/“likelihood”/“probability” of arresting/halting/restraining further breakdown of periodontal tissues.

Dental professionals should prioritize presenting clinical findings of local and general causal factors rather than discussing probability estimates generated from group data. Moreover, invoking the word “risk” in a dialogue with a patient may be misunderstood that there are elements of uncertainty on expected treatment outcomes and possible general health consequences. The clinician needs to have a clear mindset and a receptive cognitive patient to transfer the message that the uncertainty relative to “risk” lies not in an incomplete examination or uncertain diagnosis, but rather because it is merely challenging to predict the outcomes of any treatment. Alternatively, do not invoke the term “risk” in the dialogue with the patient, but focus instead on using the descriptors good, uncertain, or guarded prognosis. Moreover, in the context of the new classification, the clinician should provide specific reasons based on actual clinical findings rather than referring to abstruse epidemiological‐statistical concepts.

The rhetoric about the potential merits and disadvantages of classifying periodontitis according to staging and grading seems to be an excellent example for those interested in ordinary language philosophy and logical positivism. It is an amusing thought whether Ludwig Wittgenstein, if he still had been alive, would have labelled our collective attempts to apply a word such as “risk” independently of its context, usage, and grammar as another occurrence of language gone on holiday.
